# Ultrasound mediated delivery of oxygen and LLL12 loaded stimuli responsive microdroplets for the treatment of hypoxic cancer cells

**DOI:** 10.1038/srep44908

**Published:** 2017-03-21

**Authors:** Jinshun Xu, Shuai Yuan, Jilai Tian, Kyle A. Martin, Jinhua Song, Chenglong Li, Zhigang Wang, Jiayuh Lin, Ting Si, Ronald X. Xu

**Affiliations:** 1 Department of Biomedical Engineering, The Ohio State University, Columbus, OH, 43210, USA; 2 Chongqing Key Laboratory of Ultrasound Molecular Imaging, Second Affiliated Hospital of Chongqing Medical University, Chongqing, 400010, China; 3 School of Engineering Science, University of Science and Technology of China, Hefei, Anhui, 230026, China; 4 Center for Childhood Cancer, The Research Institute at Nationwide Children’s Hospital, Columbus, OH, USA; 5 Department of Mechanical Engineering, The Ohio State University, Columbus, OH, 43210 USA; 6 Division of Medicinal Chemistry and Pharmacology, College of Pharmacy, The Ohio State University, Columbus, OH, 43210 USA; 7 Department of Medicinal Chemistry, College of Pharmacy, University of Florida, Gainesville, FL 32610, USA

## Abstract

LLL12 exhibits high specificity for inhibiting STAT3 phosphorylation and dimerization, and inducing apoptosis to constitutively activated STAT3 cancer cells without cytotoxicity to normal cells with dormant STAT3. However, clinical deployment of LLL12 in cancer treatment is hindered by its low bioavailability and hypoxia-induced resistance. To overcome these limitations, we encapsulate both oxygen and LLL12 in stimuli responsive microdroplets (SRMs) by a gas-driven coaxial flow focusing (CFF) process for ultrasound mediated treatment of hypoxic cancer cells. Our benchtop experiments demonstrate that the CFF process is able to produce SRMs with uniform size distribution, large oxygen loading capacity, high LLL12 encapsulation efficiency, well protection of bioactivity, and steadily long shelf time. The *in vitro* therapeutic studies in pancreatic cancer cells (PANC-1 and CAPAN-1) demonstrate the immediate release of oxygen and LLL12 in exposure to therapeutic ultrasound pulses as well as the improved anticancer effects under hypoxic conditions. The findings suggest that the proposed oxygen and LLL12 loaded SRMs provide a promising drug delivery strategy for more effective treatment of hypoxic cancer cells.

Pancreatic adenocarcinoma is one of the most lethal solid tumors and the third leading cause of cancer-related death in North America^1^. Owning to the poor diagnosis of pancreatic cancer nowadays, more than 80% of patients with the locally advanced or metastatic diseases have to accept chemotherapy or radiotherapy as their primary treatment options^2^,^3^. However, these therapies do not show significant survival advantages but instead develop various complications and side effects^3^. Recently, targeted delivery of disease-specific small molecule inhibitors has demonstrated anticancer potential without significant cytotoxicity to normal cells^4^. With a structure-based drug design strategy, we have synthesized LLL12 (chemical structure is shown in Fig. 1a), a novel small molecule inhibitor with molar mass of 303.02 g/mol. This agent induces apoptosis in a variety of human cancer cells expressing signal transducer and activator of transcription (STAT3) by inhibiting activation to STAT3 signaling^4^,^5^,^6^. However, clinical efficacy of this promising therapy in pancreatic cancer treatment is still suboptimal, due to many limitations such as the poor bioavailability and the development of hypoxia-induced resistance. On the one hand, like many small molecule drugs, LLL12 has poor aqueous solubility, non-specific interactions during delivery, and can be easily metabolized to its limited bioavailability^7^. On the other hand, hypoxia in pancreatic cancer promotes tumor invasion and metastasis, reduces therapeutic efficacies for many drugs that rely on reactive oxygen species for cytotoxicity, and induces therapeutic resistance^8^.

To overcome the above limitations and improve the efficacy for pancreatic cancer treatment, we propose to encapsulate LLL12 and oxygen in stimuli-responsive microdroplets (SRMs) for ultrasound mediated controlled release at the disease site. For this purpose, a novel gas-driven coaxial flow focusing (CFF) process is used to produce multi-layered microdroplets. The CFF process forces a continuous phase of coaxial liquid flow through an orifice in a surrounding high-speed gas stream, as sketched in Fig. 1b. Since the process is based on the physical principle of three-dimensional flow focusing technique^9^,^10^,^11^, it is convenient to produce microdroplets with multiple components encapsulated in the same shell^12^,^13^,^14^. The CFF process is able to produce relatively uniform core-shell structured microdroplets with high encapsulation efficiency, long term stability, and low cost, superior to other commonly used microencapsulation processes such as emulsification^15^.

To demonstrate the ability of SRMs for ultrasound mediated treatment of pancreatic cancer PANC-1/CAPAN-1 cells, we use the CFF process to produce LLL12 loaded microdroplets (LMs) and oxygen and LLL12 loaded microdroplets (OLMs), respectively. First, both LMs and OLMs are fabricated and their size distribution, stability, LLL12 encapsulation efficiency (EE) and loading capacity (LC) are characterized. Second, ultrasound mediated release of oxygen and LLL12 from the OLMs is validated in a benchtop setup quantitatively. Further, bioavailability protection of LLL12 by microencapsulation is demonstrated in a simulated metabolic environment. Finally, ultrasound mediated treatment of pancreatic cancer cells under hypoxic condition is demonstrated and compared with conventional treatments. To the best of our knowledge, simultaneous encapsulation of LLL12 and oxygen for the treatment of hypoxic pancreatic cancer cells have not been reported elsewhere.

## Results

### CFF parameter control and SRM characterization

CFF is a purely physical process involving the mechanism of jet instability^9^,^10^,^11^,^12^,^13^,^14^. Therefore, it is technically feasible to produce monodisperse microdroplets reliably by accurate control of the process parameters. As the flow rate of the inner phase decreases or the flow rate of the focusing phase increases, the produced microdroplets demonstrate smaller size and less uniformity (Fig. 2a–b). In comparison, the flow rate of the outer phase has less influence on the size of droplets since the inner phase is encapsulated within a thin layer of lipid and the solvent of the outer phase is extracted by the solution in the collector. In this case, the outer phase provides a uniformly surrounding liquid flow for the inner phase as long as its flow rate is much greater than that of the inner phase, ensuring the formation of a consistently stable cone-jet configuration.

In this study, we choose an inner phase flow rate of 3 ml/h, an outer phase flow rate of 50 ml/h, and a focusing phase pressure difference of 9 kPa in order to optimize the size and the uniformity of the produced SRMs. The stimuli responsive capability of SRMs is related to the Laplace pressure Δ*P* of microdroplet surface given as Δ*P* = *P*_inside_ – *P*_outside_ = 2*γ*/*R*, where *γ* is the surface tension, *R* the droplet radius, *P*_inside_ the pressure inside a droplet, and *P*_outside_ the pressure outside a droplet. It can be easily seen that the Laplace pressure is heavily dependent on the droplet size. Therefore, the SRMs with relatively uniform size distribution would be well controlled for the significant stability and long shelf time. More importantly, it would offer more sufficient sensitivity and accuracy for the ultrasound-mediated drug delivery, compared to the polydisperse SRMs with broad size distribution. The synthesized OLMs have a size distribution of 4.54 ± 2.05 μm and a concentration of 3.4 ± 0.62 × 10^8^ /ml. In comparison, the size distribution of the synthesized LMs is 4.91 ± 1.45 μm and the concentration is 3.1 ± 0.35 × 10^8^/ml, indicating that loading oxygen does not induce significant morphological differences (Fig. 2c–f). The size and the concentration of the microdroplets show no significant variation for at least seven days at both 4 °C and 37 °C, demonstrating the high stability for both OLMs and LMs (Fig. 2g–j). The droplet stability is mostly dependent on the physical and physicochemical properties of lipid shell and PFC core. Considering that oxygen is dissolved in the PFC and LLL12 solution, its contribution to droplet stability is negligible as long as the phase shift does not occur. In this case, OLMs and LMs with relatively narrow size distribution could be transported freely into pancreatic cancer regions via blood circulation without pulmonary embolism and thrombosis. Simultaneously, the preferential uniformity could ensure the effective vaporization of OLMs under ultrasound mediation to achieve the improvement of drug and oxygen release for anticancer treatment.

### Encapsulation and controlled release of OLMs and LMs

Using the CFF process, we are able to encapsulate LLL12 in OLMs and LMs at high EE levels of 94.56 ± 4.88% and 94.03 ± 5.74%, respectively (Fig. 3a). The LC levels of OLMs and LMs are 15.40 ± 2.07% and 16.68 ± 3.45%, respectively (Fig. 3b). The drug release kinetics from the LMs and the OLMs are measured at 37 °C over a long period with and without ultrasound irradiation. As shown in Fig. 3c, the initial LLL12 release from LMs without ultrasound irradiation is as low as ~6% over 8 h. Upon ultrasound irradiation at 2 h, a significant amount (~80%) of LLL12 is rapidly released within less than 1 h. Similar trends are also observed for the LLL12 release from OLMs (Fig. 3d). These results indicate that oxygen loading has little effect on LLL12 encapsulation and release characteristics of the produced microdroplets.

Oxygen release kinetics are also determined by suspending OLMs in PBS at 37 °C. Without ultrasound irradiation, the suspension shows little variation of the dissolved oxygen as monitored for 3 mins (Fig. 3e), indicating that oxygen is effectively retained in OLMs without leakage. After OLMs are destructed by ultrasound pulses, the dissolved oxygen is increased by more than 40% in 6 mins. In comparison, the control sample of LMs does not show significant increase of the dissolved oxygen after ultrasound irradiation. These results demonstrate that ultrasound irradiation of the OLMs can effectively increase the dissolved oxygen *in situ*.

In addition, the bioavailability of LLL12 is further determined in Fig. 3f. Without encapsulation, more than 50% of LLL12 changes its spectral characteristics after interacting with microsome for 0.5 h. In comparison, encapsulating LLL12 in OLMs and LMs protects more than 50% of LLL12 from molecular interaction after incubation with microsome for 2 h. These results demonstrate that microencapsulation effectively protects LLL12 from non-specific interactions and significantly enhances its bioavailability.

### Anticancer potency

Ultrasound mediation of colony formation assay is used to determine the anticancer potency of OLMs and LMs. After co-culturing PANC-1 cells with media at different treatment groups under 21% oxygen for 14 days, the treatment groups of LLL12, LMs and OLMs yield a significantly lower number of colonies in comparison with control treatments of either DMSO or blank microdroplets only (Fig. 4a). Further quantitative analysis shows no significant difference in colony formation among the treatment groups of LLL12, LMs and OLMs (P > 0.05, Fig. 4b), indicating that microencapsulation processing of drugs has little effect on the anticancer potency of LLL12. Under 1% oxygen, the same LLL12 dose (0.5 μM) cannot effectively inhibit colony formation of PANC-1 cells in the LLL12 and LMs groups due to the development of hypoxic-induced chemoresistance (Fig. 4a). However, few colony is formed after the treatment of OLMs, indicating strong anticancer potency in the hypoxic PANC-1 cells. Further quantitative analysis demonstrates the ultrasound mediated release of oxygen from OLMs, the increased oxygen in microenvironment, and the reversed chemoresistance for LLL12 treatment of hypoxic pancreatic cancer PANC-1 cells, as shown in Fig. 4c.

The above experiment is repeated in CAPAN-1, another pancreatic cancer cell line, in order to verify the anticancer potency of OLMs. According to the experimental results shown in Figs. 4d,e, both LMs and OLMs show strong anticancer potency under 21% oxygen while only OLMs show strong anticancer potency under 1% oxygen, indicating that co-delivery of oxygen and LLL12 in SRMs enhances the anticancer potency of LLL12 in the treatment of hypoxic pancreatic cancer CAPAN-1 cells.

## Discussion

Known as one of the most lethal solid tumors in the world, pancreatic cancer has poor diagnosis and frequent relapse^16^. Despite several decades of research efforts, few curative treatments have been developed for this life-threatening disease^1^. The poor outcome is mainly due to the low bioavailability and the development of resistance for many anticancer therapies^17^. Fortunately, the significant antitumor activity of LLL12 through directly inhibiting STAT3 activation has been reported recently in a variety of cancer cells^4^,^5^,^6^. However, further antitumor activity of this therapy in the pancreatic cancer cells was hindered by its poor bioavailability^7^ as well as hypoxia-induced resistance^8^,^18^. Therefore, enhancing drug delivery efficiency and increasing oxygenation at the disease site become necessary. Recently, monodisperse microdroplets have been fabricated by microfluidic techniques for the enhanced drug delivery efficiency in cancer therapy^19^,^20^,^21^. These advances enable us to produce oxygen and LLL12 loaded SRMs by the CFF process and test the hypothesis that ultrasound mediation of these SRMs will increase drug and oxygen deposition at the tumor site for the enhanced therapeutic outcome.

Our SRM agent has a lipid membrane and a core of PFC in liquid phase. Oxygen and LLL2 are encapsulated in the SRM agent for ultrasound mediated release of combinatory therapeutics. Compared to the conventional microbubbles with a gas core exhibiting a short life time of 2 days at room temperature^22^, the produced SRMs (both OLMs and LMs) exhibit more significant stability at both room and body temperatures for more than 7 days. The extended life time of the SRM agent may be attributed to its liquid core that reduces the molecular diffusion among microdroplets^23^. For both OLMs and LMs, the CFF process achieves LLL12 EE levels of above 94%, much higher than that of a conventional emulsification process. After applying therapeutic ultrasound pulses of 1 MHz at an intensity of 3 W/cm^2^ and a duty cycle of 20% for the duration of 2 min, both OLMs and LMs release over 80% of the loaded drugs within less than 1 h. Simultaneously, oxygen is also released from the OLMs for reversed chemoresistance and enhanced anticancer potency, as suggested in previous works^24^,^25^,^26^.

Based on our preliminary study, an LLL12 dose of 0.5 μM for PANC-1 cells and 1.0 μM for CAPAN-1 cells induce significant anticancer activities in 21% oxygen but not in 1% oxygen. Therefore, it is appropriate to use these cancer cells to study hypoxia-induced chemoresistance. Therapeutic ultrasound pulses induce significant inhibition of cell proliferation for treatment groups of not only LMs and OLMs in 21% oxygen, but also OLMs in 1% oxygen. The possible causes of the enhanced anticancer potency for LLL12 in presence of oxygen could be multifactorial. First, as a characteristic feature of many locally advanced solid tumors, hypoxia enhances drug resistance of cancer cells through different mechanisms such as reduced cellular uptake, reduced generation of free radicals for cytotoxicity and cellular adaptations to chemotherapy^27^. Therefore, localized delivery of oxygen may boost tumor tissue oxygenation, increase its responsivity, and reverse chemoresistance. Second, applying ultrasound pulses to tumor tissue may facilitate the sonodynamic therapeutic effect that produces cytotoxic reactive oxygen species (ROS) for cytotoxicity^28^, where increased oxygen concentration may proportionally increase mitochondria production of ROS^29^. Finally, ROS further stimulates the activation of STAT3 through Src and JAK pathways, leading to potentially enhanced sensitivity and specificity for STAT3-targeting therapies^30^. Further study is needed in order to delineate the in-depth mechanism of anticancer synergy by co-delivery of oxygen and LLL12.

The ultrasound mediated synergistic effect of therapeutics is associated with the following possible mechanisms. First of all, ultrasound pulses stimulate the phase transition of PFC liquid for acoustic droplet vaporization (ADV)^31^,^32^. Second, ultrasound mediated cavitation facilitates rapid release of therapeutics, reduced time for localized drug delivery, increased drug accumulation at the disease site, and enhanced bioactivities for anticancer agents^33^,^34^,^35^. Third, ultrasound fragmentation of the microdroplets generates physical forces, shock waves, microjets, and microstreamings to increase the permeability of cell membranes for intracellular uptake of drugs^36^,^37^. Fourth, oxygen released from the SRMs provides an oxygen-enriched environment and increases sensitivity for many anticancer therapies^8^,^38^. Finally, ultrasound mediation may stimulate the generation of reactive oxygen species, such as singlet oxygen, for the enhanced cytotoxicity^39^,^40^. Our findings have established an important proof of concept for ultrasound mediated treatment of hypoxic pancreatic cancer. The next work includes engineering optimization and biologic validation. On one side, the SRM design will be optimized and the acoustic fragmentation process will be analyzed for the enhanced treatment outcome and the reduced side effects. On the other side, the therapeutic efficacy of simultaneous delivery of oxygen and LLL12 will be validated *in vivo* in a cancer xenograft model.

## Conclusions

We have encapsulated oxygen and LLL12 in SRMs by a novel CFF process for ultrasound mediated treatment of hypoxic pancreatic cancer cells. The produced SRMs have a stably long shelf life, superior bioavailability, high efficiency for LLL12 encapsulation and effective reservation of oxygen before ultrasound fragmentation. Our study reveals that the ultrasound mediated oxygen and drug delivery platform is able to accomplish an enhanced anticancer effect on PANC-1/CAPAN-1 pancreatic cancer cells under hypoxic conditions *in situ*. These results may present a promising and attractive approach for pancreatic cancer therapy.

## Methods

### Materials and Cell culture

Hydrogenated L-a-phosphatidylcholine, polyethylene glycol 40 (PEG-40) stearate, glycerol, propane-1,3-diol and Tween^®^ 80 are obtained from Sigma–Aldrich (St. Louis, MO). 1,1,1,2,3,4,4,5,5,5-decafluoro-pentane, a perfluorocarbon (PFC) compound with boiling point of 55 °C, is obtained from Fluka (St. Louis, MO). LLL12 is provided by College of Pharmacy at the Ohio State University.

Human pancreatic carcinoma PANC-1/CAPAN-1 cell lines are maintained at Cancer Research Center of Nationwide Children’s Hospital (Columbus, OH). The PANC-1 cells and CAPAN-1 cells were cultured in DMEM and RPMI1640 medium (Gibco), respectively, and supplemented with 10% fetal bovine serum (FBS) and 1% penicillin/streptomycin at 37 °C in a humidified incubator containing 5% CO_2_. Exponentially growing cells are used for all the experiments.

### Experimental setup of the gas-driven CFF

**Figure 1:**
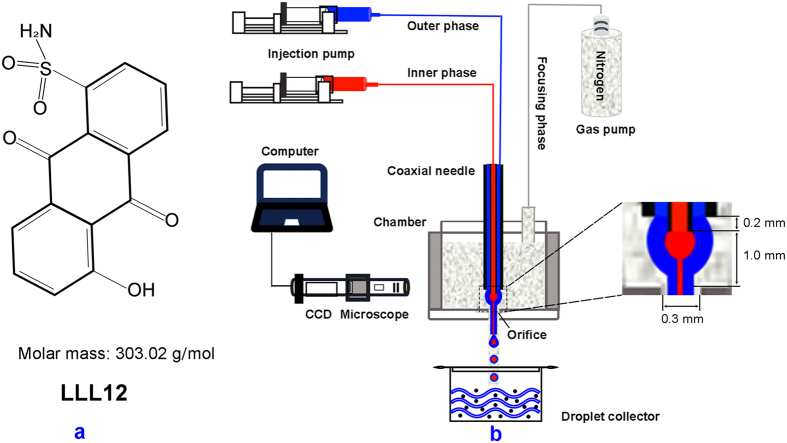
(**a**) Chemical structure of LLL12; (**b**) Schematic illustration of the gas-driven CFF setup.

**Figure 2:**
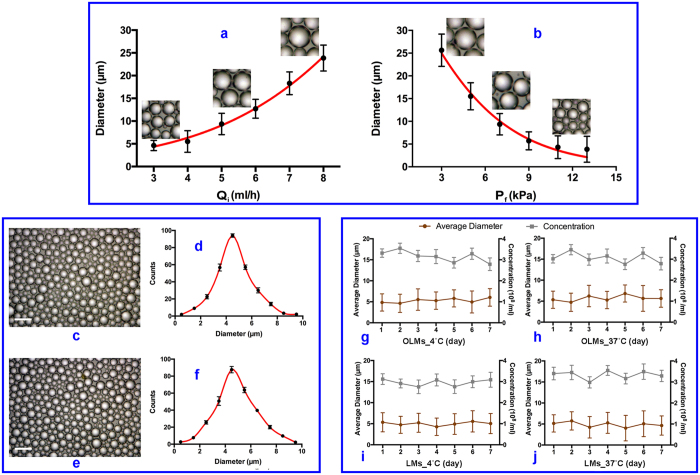
(**a**) Droplet diameter as a function of Q_i_ for P_f_ = 9 kPa, Q_o_ = 50 ml/h; (**b**) Droplet diameter as a function of P_f_ for Q_i_ = 3 ml/h, Q_o_ = 50 ml/h; (**c,d**) Microscopic image and size distribution of oxygen and LLL12 loaded microdroplets (OLMs); (**e,f**) Microscopic image and size distribution of LLL12 loaded microdroplets (LMs); (**g,h**) Size change over time for OLMs at 4 °Cand 37 °C respectively; (**i,j**) Size change over time for LMs at 4 °Cand 37 °C respectively. Error bars in data represent S.D. (n = 3).

**Figure 3:**
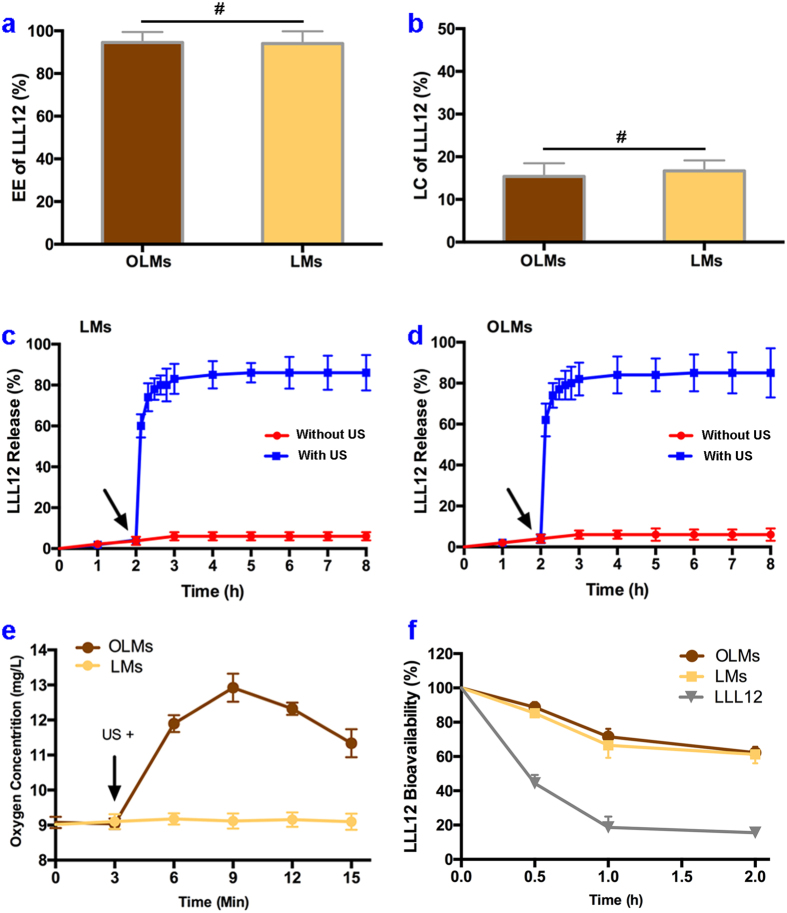
(**a**) Encapsulation efficiency (EE) and loading capacity (LC) of LLL12 in OLMs and LMs, respectively; (**c,d**) Release of LLL12 from LMs and OLMs respectively with and without ultrasound exposure. Arrows indicate the time points for ultrasound mediation; (**e**) Oxygen release from OLMs before and after ultrasound exposure at the time-point of 3 min (arrow); (**f**) Bioavailabilities of LLL12 in microsome solution with and without SRM encapsulation. ^*#*^*P* > 0.05, *P < 0.05 with others. Error bars in all data represent S.D. (n = 3).

**Figure 4:**
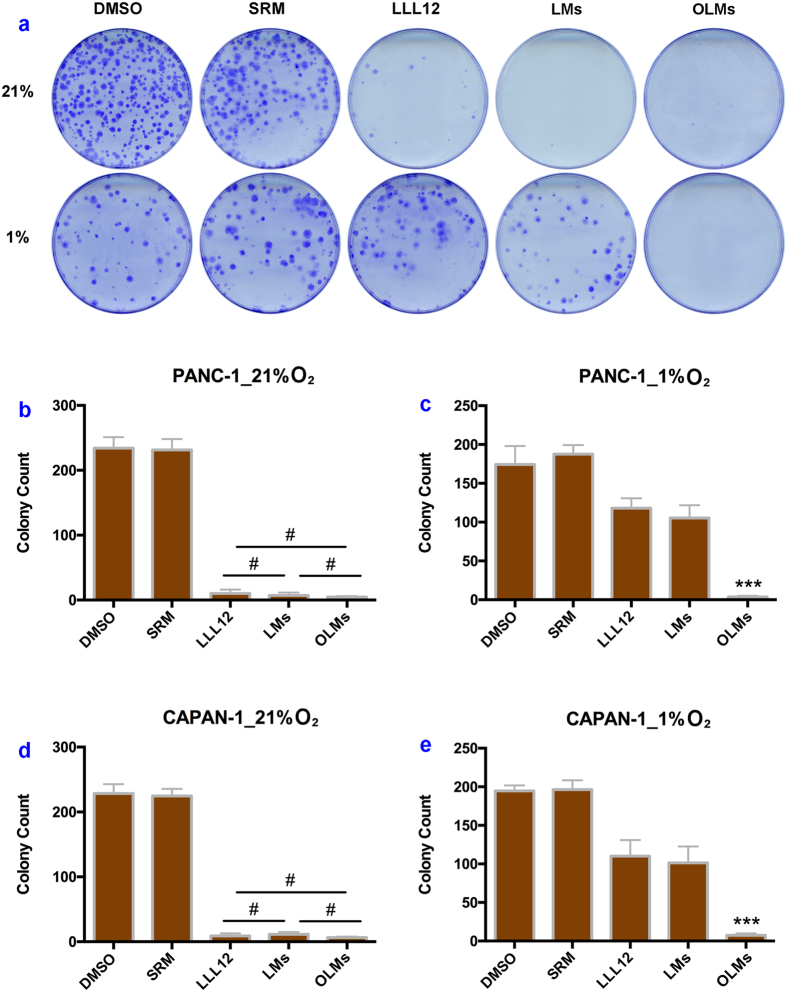
Cancer cell colony formations after different treatments at normoxic and hypoxic conditions. (**a**) PANC-1 colony photograph microscopy after treatment two weeks among different groups; (**b,c**) Analysis comparison of PANC-1 colony numbers counted 2 weeks post treatment in 21% and 1% oxygen, respectively; (**d,e**) Analysis comparison of CAPAN-1 colony numbers counted 2 weeks post treatment in 21% and 1% oxygen, respectively. ^*#*^*P* > 0.05, ***P < 0.001 versus other groups. Error bars in all data represent S.D. (n = 3).

The experimental setup for the gas-driven CFF process (i.e., Fig. 1b) includes two injection pumps, one gas pump, a coaxial needle assembly, a pressure chamber and a monitor. The coaxial needle assembly is fabricated by the laser beam welding method^14^, through inserting an inner needle (inner diameter: 0.41 mm, outer diameter: 0.71 mm) into an outer needle (inner diameter: 1.01 mm, outer diameter: 1.48 mm) for high concentricity and complete sealing. The tip of the inner needle is 0.2 mm longer than that of the outer needle. The pressure chamber (width: 19 mm, length: 25 mm) is made to fix the coaxial needle on the top using a rubber plug. An orifice (diameter: 0.3 mm) facing the exit of the needle assembly is machined on the bottom center of the chamber to adjust 1 mm of the distance from the inner needle tip to the orifice.

### Preparation and characterization of OLMs and LMs

Lipid suspension is prepared in advance by a mass ratio (1:0.28:25:105:43:1000) of mixing hydrogenated L-a-phosphatidylcholine (50 mg), PEG-40 stearate (14 mg), glycerol (1 ml, density 1.25 g/ml), propane-1,3-diol (5 ml, density 1.053 g/ml), Tween^®^ 80 (2 ml, density 1.080 g/ml), and distilled water (50 ml) in a conical flask. The mixture is placed in a 42 °C water bath to gently stir for 30 min, and then cooled down to room temperature. The LLL12 loaded PFC solution is fabricated via gently stirring the mixture of LLL12 (1 mg), DMSO (1 ml), and PFC (9 ml)for 1 h. The LLL12 and oxygen loaded PFC solution is prepared through bubbling the LLL12 loaded PFC solution (10 ml) with oxygen till saturation. The oxygen is loaded in droplets as a molecular status.

The OLMs are fabricated using the gas-driven CFF device. Two syringe pumps are used to provide continuous flow of the inner phase (flow rate Q_i_ = 3 ml/h) of PFC solution loaded oxygen and LLL12 and the outer phase (flow rate Q_o_ = 50 ml/h) of lipid suspension. A nitrogen gas cylinder is used to provide continuous flow of the focusing phase (pressure difference within the chamber P_f_ = 9 kPa). All the processes are continuously monitored using a CCD camera (Allied vision technologies, Exton, PA) equipped with a microscopic lens. The illumination is provided using a strobe flashlight (flashing frequency: 3.2 kHz) from the other side of the chamber. The obtained emulsions are centrifuged at 800 rpm for 5 min. After centrifugation, the supernatant is discarded and the precipitate is redispersed in the 2 ml of phosphate buffered saline (PBS, PH 7.4) solution for further use. The LMs are similarly prepared using LLL12 loaded PFC solutions without bubbling oxygen.

The samples from OLMs and LMs are observed in the microscope. The size distributions of microdroplets are determined using the NIS-Elements and Viewer software (Nikon, Melville, NY) and their concentrations are measured using hemocytometers.

### Characteristics of OLMs and LMs

The amount of LLL12 incorporated in the OLMs and LMs is detected by the ultraviolet-visible spectrophotometer (UnicoS2150UV, Eray Medical, Holbrook, NY) based on absorbance at 405 nm. The EE and LC of drugs are calculated by the following equations:


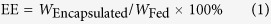



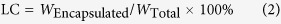


where *W*_Encapsulated_ represents the amount (in weight) of LLL12 incorporated into microdroplets and *W*_Fed_ is the amount of LLL12 initially fed for encapsulation and *W*_Total_ is the total amount of microdroplets including LLL12 and PFC and lipid materials for the microencapsulation.

### Ultrasound mediated drug and oxygen release

To determine the drug release, OLMs or LMs of 0.5 ml (~2.1 × 10^8^/ml) are injected into PBS of 4.5 ml, and transferred into dialysis tubes (MWCO: 20 kDa), and incubated in PBS of 30 ml at 37 °C under mild agitation in a water bath. At various times, the dialysate of 200 μl is collected and the remaining dialysate is replenished with the same amount of fresh PBS. The concentration of the released LLL12 in the removed dialysate is determined using ultraviolet–visible spectrophotometry based on absorbance at 405 nm. For ultrasound mediated drug release, the microdroplet solutions are sonicated for 2 min at a power density of 3.0 W/cm^2^ and a duty cycle of 20% with a frequency of 1 MHz. Then the supernatants are obtained by centrifuging at 800 rpm for 5 min to analysis using ultraviolet–visible spectrophotometry in the same way.

To determine the oxygen release, OLMs of 0.5 ml (~2.1 × 10^8^/ml) is injected into PBS of 4.5 ml in the 37 °C water bath. The concentration of dissolved oxygen is measured over 15 min using a dissolved oxygen meter (DO210, Extech Instruments, Nashua, NH). After 3 min, ultrasound is exposed for 2 min using the same parameters mentioned above. The values for the control are obtained using LMs without oxygen following the same procedure.

### Protection of LLL12 bioavailability by microdroplet encapsulation

To determine the protection of LLL12 bioavailability by microdroplet encapsulation, OLMs or LMs of an amount 10 μl (LLL12: 0.5 mg/ml) are injected into microsome of 0.5 ml (0.5 mg/ml). After adding 10 μl DMSO at the time point of 0 h, 0.5 h, 1 h, 2 h, respectively, ultraviolet-visible absorption is detected at 405 nm. The same dose of the blank microdroplets without loading LLL12 and DMSO is also injected into microsome for baseline determination. The LLL12 concentrations of OLMs and LMs are calculated based on the absorption using a standard curve of LLL12 solutions (5, 10, 15 and 20 μg/ml in microsome). The same does of LLL12 only without microdroplet encapsulation is also performed following the same procedure as controls. All measurements are carried out in triplicate.

### Ultrasound mediated colony formation assay

Two pancreatic cells (2 × 10^5^ cells per well) including PANC-1 and CAPAN-1 are incubated with medium (3 ml per well) in six-well plates for 24 h. When the specimens become 80% confluent, LLL12 of 0.5 μM (in PANC-1 cells) or 1.0 μM (in CAPAN-1 cells) is added into the wells in three cases, including OLMs, LMs, and LLL12 only, for 12 h at 21% or 1% oxygen conditions, respectively. The same does of microdroplets only and DMSO is also added as controls. In the 1% oxygen chamber, each well is sealed using a processed robber plug to cut off the air. To release drugs and oxygen from microdroplets, ultrasound is exposed in advance for 2 min with the parameters mentioned above in OLMs, LMs, and microdroplets only groups. After incubation for 12 h, the specimens are trypsinized. Viable cells are counted and then plated at a density of about 1000 cells per dish with 6 cm in diameter. The specimens are incubated at 37 °C for 2 weeks for colony formation. Each colony is stained for 4 h by crystal violet dye (2 ml per dish) using a shaker. Lastly, photographs of any colonies in every dish are taken using a color image scanner (Canon, Melville, NY). The number of colonies in each dish is scored for quantify analysis and comparison. All measurements are carried out in triplicate.

### Statistical Analysis

Experimental data presented as mean ± standard deviation (SD) are analyzed using SPSS 17.0 software. Multiple comparisons are performed by one-way ANOVA analysis followed by the Tukey post hoc test. Differences are considered statistically significant when *P < *0.05 with 95% confidence intervals.

## Additional Information

**How to cite this article**: Xu, J. *et al*. Ultrasound mediated delivery of oxygen and LLL12 loaded stimuli responsive microdroplets for the treatment of hypoxic cancer cells. *Sci. Rep.* **7**, 44908; doi: 10.1038/srep44908 (2017).

**Publisher's note:** Springer Nature remains neutral with regard to jurisdictional claims in published maps and institutional affiliations.
